# Nonoperative management of blunt renal trauma: Is routine early follow-up imaging necessary?

**DOI:** 10.1186/1471-2490-8-11

**Published:** 2008-09-03

**Authors:** John B Malcolm, Ithaar H Derweesh, Reza Mehrazin, Christopher J DiBlasio, David D Vance, Salil Joshi, Robert W Wake, Robert Gold

**Affiliations:** 1 Department of Urology, University of Tennessee Health Science Center, Memphis, Tennessee, USA; 2 Department of Radiology, University of Tennessee Health Science Center, Memphis, Tennessee, USA; 3 Urology Service, Elvis Presley Memorial Trauma Center, Regional Medical Center at Memphis, Tennessee, USA

## Abstract

**Background:**

There is no consensus on the role of routine follow-up imaging during nonoperative management of blunt renal trauma. We reviewed our experience with nonoperative management of blunt renal injuries in order to evaluate the utility of routine early follow-up imaging.

**Methods:**

We reviewed all cases of blunt renal injury admitted for nonoperative management at our institution between 1/2002 and 1/2006. Data were compiled from chart review, and clinical outcomes were correlated with CT imaging results.

**Results:**

207 patients were identified (210 renal units). American Association for the Surgery of Trauma (AAST) grades I, II, III, IV, and V were assigned to 35 (16%), 66 (31%), 81 (39%), 26 (13%), and 2 (1%) renal units, respectively. 177 (84%) renal units underwent routine follow-up imaging 24–48 hours after admission. In three cases of grade IV renal injury, a ureteral stent was placed after serial imaging demonstrated persistent extravasation. In no other cases did follow-up imaging independently alter clinical management. There were no urologic complications among cases for which follow-up imaging was not obtained.

**Conclusion:**

Routine follow-up imaging is unnecessary for blunt renal injuries of grades I-III. Grade IV renovascular injuries can be followed clinically without routine early follow-up imaging, but urine extravasation necessitates serial imaging to guide management decisions. The volume of grade V renal injuries in this study is not sufficient to support or contest the need for routine follow-up imaging.

## Background

Nonoperative management has become the rule for the majority of blunt renal injuries, with higher rates of renal salvage and decreased morbidity compared to primary surgical management [1]. The nonoperative management scheme is not standardized amongst all urologists, but typically involves a period of bed rest, monitoring of vital signs and serial hematocrit measurements, with either selective or routine use of early follow-up imaging. Our center has previously advocated routine follow-up imaging 2 to 4 days after blunt renal trauma to identify patients that may require intervention for delayed complications [2]. However, noting in recent years that the vast majority of follow-up CT scans do not alter clinical management, we have elected to reevaluate our previously proposed management strategy. We reviewed our contemporary experience with nonoperative management of blunt renal injuries in order to reassess the utility of routine early follow-up imaging.

## Methods

After receiving approval from the Institutional Review Board of the University of Tennessee Health Science Center, Memphis, Tennessee, we performed a retrospective chart review of all patients admitted with blunt renal injury and primary nonoperative management at the Elvis Presley Memorial Trauma Center between 1/2002 and 1/2006. Data collected from chart review included patient age and gender, race, body mass index (BMI, kg/m^2^), Glascow coma score (GCS), mechanism of injury, side of injury, grade of injury, vital signs, serial hematocrit measurements, results of follow-up imaging, complications, and delayed interventions.

All injuries were diagnosed at the time of admission using contrasted CT imaging in the cortical and delayed excretory phases. Imaging for the diagnosis of renal trauma was obtained based on standard indications for the adult trauma patient: gross hematuria, microscopic hematuria with hypotension, or high suspicion of renal injury based on the mechanism of trauma [3,4]. Injuries were graded by a staff radiologist according to the American Association for the Surgery of Trauma (AAST) organ injury scale [5]. Injuries were also independently evaluated and graded by the managing urologist. Where discrepancies in grading were noted on chart review, the imaging studies were reread to verify accurate injury grading. However, all films were not uniformly reread at the time of chart review.

Our renal trauma database captured all patients admitted with blunt renal injury and primary nonoperative management. It did not include the rare patient who underwent primary operative management or those with grade I injuries that were deemed appropriate for outpatient management by the trauma service. Patients who were hemodynamically stable at the time of presentation were managed according to a nonoperative protocol with bed rest, serial measurement of vital signs and hematocrit every 6 hours until stable over a 24-hour period or until gross hematuria resolved, and follow-up CT imaging 24–48 hours after admission.

After compiling data from chart review, we noted the rate of clinically significant new findings on repeat imaging and attempted to correlate clinical outcomes with repeat imaging results. Student's t-test was used to compare demographic subsets in our series. Fisher's exact test was used to compare re-imaging outcomes between patients with low-grade (I,II,III) and high-grade (IV,V) injuries.

## Results

Patient demographics are shown in Table 1. 207 patients (mean age 35 years, 120 male/87 female) were admitted for nonoperative management of 210 blunt renal injuries (3 bilateral) between 1/2002 and 1/2006. Table 2 shows radiographic findings and clinical outcomes. AAST grades I, II, III, IV, and V were assigned to 35 (16%), 66 (31%), 81 (39%), 26 (13%), and 2 (1%) renal units, respectively. Among grade IV injuries, 19 (73%) were renovascular injuries (segmental infarcts) and 7 (27%) involved collecting system injury (urinary extravasation). Average BMI among patients with low-grade injury (grades I, II, and III) was 26.6, compared to 27.0 among patients with high-grade injury (grades IV and V) (p = 0.81). 177 (84%) renal injuries underwent routine follow-up imaging 24–48 hours after admission. Among the 33 (16%) renal injuries that were not re-imaged, 17 (52%), 9 (27%), and 6 (18%) were of injury grades I, II, and III, respectively. One patient with a grade V renal injury was not stable enough for transport to radiology for follow-up imaging, and he ultimately succumbed to multiple traumatic injuries.

**Table 1:** Demographics and clinical presentations

Variable	
Number of patients	207
Age (years)	
Mean (range)	35 (15–80)
BMI (kg/m^2^)	
Mean (range)	26.7 (17.8–45.6)
Gender	
Female	87 (42%)
Male	120 (58%)
Glascow Coma Score (GCS)	
Mean (range)	13.6 (3–15)
Mechanism of Injury	
MVA	173 (84%)
Pedestrian Struck	15 (7%)
Fall	13 (6%)
Assault	6 (3%)
Race	
African American	79 (38%)
Caucasian/other	128 (62%)
Side of Injury	
Left	108 (52%)
Right	96 (46%)
Bilateral	3 (2%)

**Table 2:** Radiographic and clinical outcomes

	Grade	N (%)	F/U Imaging	Injury D/G*	Injury U/G**	%D/G*	%U/G**	Complications	Interventions
Low Grade	I	35 (16)	19 (54%)	4 (21%)	0	12%	3%	0	0
	II	66 (31)	57 (86%)	5 (9%)	2 (4%)			0	0
	III	81 (39)	75 (93%)	9 (12%)	2 (3%)			1 (1%)	0

High Grade	IV	26 (13)	25 (96%)	1 (4%)	0	4% P = 0.32***	0 P = 1.00***	3 (12%)	3 (endoscopic ureteral stent)
	V	2 (1)	1 (50%)	0	0			1 (50%)	0

	Total	210	177 (84%)	19 (11%)	4 (2%)			5 (2.4%)	3 (1%)

* D/G = Downgrade

** U/G = Upgrade

*** Fisher's exact test

We noted low rates of altered injury grading after follow-up imaging. After early re-imaging, renal injuries were downgraded in 4 (21%), 5 (9%), 9 (12%), 1 (4%), and 0 cases of grade I, II, III, IV, and V injury, respectively. Grade I injuries were downgraded when subcapsular hematoma was not evident on follow-up imaging; higher grade injuries were downgraded when lacerations appeared smaller or fewer in number on follow-up imaging compared to initial imaging. Renal injuries were upgraded in 0, 2 (4%), 2 (3%), 0, and 0 cases of grade I, II, III, IV, and V injury, respectively. Overall, the rate of injury downgrading was 12% for low-grade injury and 4% for high-grade injury (p = 0.32). The rate of injury upgrading was 3% for low-grade injuries and 0% for high-grade injuries (p = 1.00). There was no significant difference in the rates of altered injury grading on follow-up imaging between low and high-grade injuries.

Of note, two cases of grade III injury were upgraded to grade IV on follow-up imaging. In the first case, the initial CT was performed with suboptimal delayed excretory phase imaging; urinary extravasation that was not apparent on the initial CT was demonstrated on the follow-up CT with appropriately timed delayed excretory phase imaging. The second case of grade III injury upgrade involved a patient with two devascularized segments on follow-up imaging in addition to a stable 1.5 cm laceration noted on the initial CT scan. This patient was managed without surgical intervention, and there were no delayed urologic complications.

Complications and delayed interventions were uncommon in this series. In three cases of grade IV renal injury with collecting system insult, a ureteral stent was placed after serial imaging demonstrated persistent extravasation; endoscopic management proved definitive in these patients. One patient with a grade III renal injury developed a febrile urinary tract infection that was successfully managed with IV antibiotics. There were no cases in which repeat imaging results independently prompted urologic intervention. There were no urologic complications among cases for which follow-up imaging was not obtained.

## Discussion

The incidence of traumatic renal injuries in the United States is approximately 5 per 100,000 persons [6], or 15,000 per year nationwide. The majority of renal injuries can be managed nonoperatively, with few absolute indications for surgical intervention [7]. CT imaging results factor prominently in the initial management strategy for blunt renal trauma, allowing for reliable injury grading that has been shown to correlate well with the need for surgical intervention [8,9]. However there is little consensus on the role of routine re-imaging once a nonoperative management course has been selected.

Our institution previously reported a retrospective review of 48 cases of blunt renal injury and primary nonoperative management, noting that one in ten patients with a grade II or higher blunt renal injury had a delayed urologic complication detected by follow-up CT scan that ultimately required invasive intervention [2]. Following publication of our previous institutional experience, we have maintained a protocol of nonoperative management that includes routine re-imaging of all blunt renal injuries 24–48 hours after admission. We elected to reevaluate this protocol because in our contemporary experience it has seemed that few, if any, routine re-imaging studies have independently altered clinical management. At a cost of approximately $700.00 per imaging evaluation (based on Medicare 2005 reimbursement rate for CT abdomen w/wo contrast [74170] and CT pelvis w/wo contrast [72194]), more selective use of CT imaging in the nonoperative management of blunt renal trauma could offer substantial cost-containment benefit. In the series presented, routine use of early re-imaging amounted to a cost of $121,800 (174 × $700), which proved to be, by and large, an unnecessary expense. If early re-imaging had been used selectively (only grade IV collecting system injuries and grade V injuries), as is our current practice, the cost would have been $7700 (11 × $700), realizing a cost reduction of almost 94%. Furthermore, the clinical benefit of reducing unnecessary radiation exposure is likely to be significant.

Our contemporary retrospective review includes 175 patients (177 renal units) who underwent routine early follow-up imaging during nonoperative management of a blunt renal injury. The majority of these renal units (151/85%) suffered a grade I, II, or III injury. It is noteworthy that the proportion of grade I injuries was significantly smaller than other published blunt renal trauma series (16% vs. 64% [6] and 86% [8]). It is probable that a significant proportion of patients with grade I renal injuries were deemed appropriate for outpatient management by the trauma surgery service, and were therefore not captured in our database. Among patients with low-grade renal injury, there were no instances where early re-imaging detected or prevented a urologic complication. Of some concern, a single patient was found to have urinary extravasation on follow-up imaging not appreciated on initial CT. However, in this case the initial CT scan was of suboptimal diagnostic quality due to poorly timed delayed excretory phase imaging. This illustrates the importance of high-quality imaging from the outset of patient management, particularly in a management scheme that excludes routine early re-imaging. Nevertheless, after demonstration of limited urinary extravasation on follow-up imaging, this patient was managed nonoperatively and additional imaging 5 days later revealed resolution of the urine leak.

The goals of nonoperative management of blunt renal injury are to identify, manage, and limit associated complications – including urinary extravasation, urinoma, infection, bleeding, and, most importantly, loss of renal function or unnecessary nephrectomy. Such complications have been reported in 3% to 33% of patients after renal trauma [10]. Clinical management of such complications is directed primarily by objective clinical signs and symptoms (i.e., hemodynamic instability, increasing pain, fever and leukocytosis, decreasing hematocrit and blood transfusion requirement) and not by imaging results [11]. Even in cases where imaging results demonstrate known harbingers of urologic complications (devascularized segments, urinary extravasation), continued nonoperative management has proven practicable, with intervention based on clinical rather than radiographic criteria [11]. It is our contention that the optimum screening protocol for urologic complications in nonoperatively managed blunt renal injury should rely primarily on objective clinical signs and symptoms to the exclusion of routine, repeat, radiographic imaging.

Our series of 207 patients (210 renal units) includes 32 patients (33 renal injuries) who did not undergo repeat imaging. The majority of patients in this subset had a grade I or grade II injury that was managed by the trauma surgery service without consultation by the urology service. Excluding one patient with a grade V renal injury (early mortality), there were no urologic complications among these patients. Admittedly, this group has limited statistical significance given its diminutive power.

**Figure 1:**
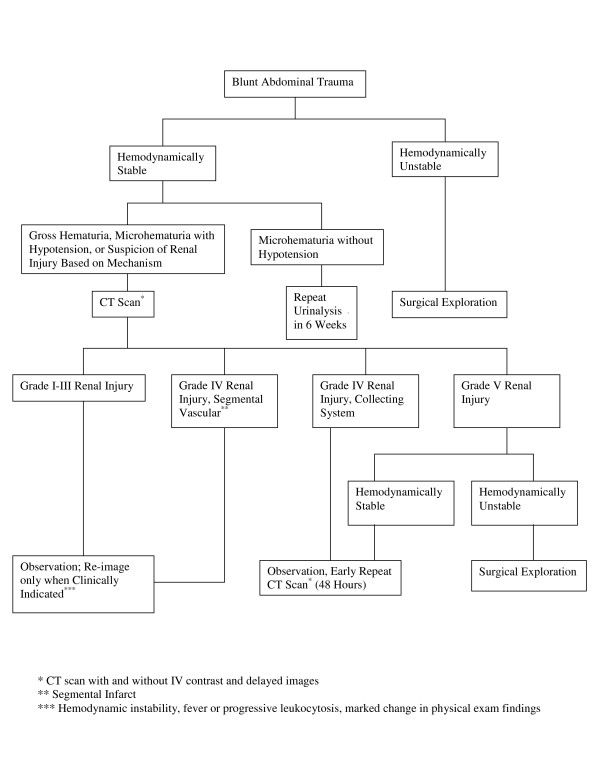
Blunt renal injury management algorithm.

We are prospectively evaluating a revised management strategy (Figure 1), and future study will test our current conclusion that routine re-imaging of grade I-IV renal injuries is unnecessary. Since reviewing our experience with blunt renal trauma management from 2002 to 2006, we have abandoned routine early re-imaging for blunt renal injuries of grades I-III and grade IV renal injuries without urinary extravasation. We now use re-imaging studies selectively for patients with grade IV injuries with demonstrated urinary extravasation, patients with multiple comorbidities who are putatively at increased risk for complications from renal trauma, patients with severe injuries involving multiple organ systems, and patients with clinical signs (hemodynamic instability, decreasing hematocrit, fever) that may herald progressing complications from blunt renal injury. We continue to routinely re-image the rare patient who meets criteria for nonoperative management of a grade V renal injury. Our experience with this management algorithm will be reported as a sizeable experience accrues.

Weaknesses of this study include its retrospective design, with the inherent limitations and biases of a retrospective analysis. Furthermore, we do not have long-term follow-up data for the majority of the patients in this cohort, so we are unable to evaluate the impact of routine re-imaging on long term renal functional outcomes, development of hypertension, or other renal injury sequellae. We suspect that the impact of routine re-imaging on such parameters is minimal. Additionally, we have reviewed the use of routine re-imaging 24–48 hours after blunt renal injury. It has been shown that many of the delayed complications from blunt renal trauma (delayed bleed, AVF, infected urinoma, abscess) occur at least 1–3 weeks after the injury occurs [7], so it is possible that routine re-imaging of blunt renal injuries would yield more clinically useful results if performed at a longer time-interval post injury, i.e. 1–3 weeks. Ultimately, we feel that such a management scheme is not practicable, and if 2–3 week follow-up is achievable we feel that more cost-effective and efficient screening for delayed complications can be achieved by physical exam, vital signs, and simple laboratory tests (hematocrit and serum creatinine). One additional complication of this study lies in the grading system used for blunt renal injuries. The AAST renal injury scale is straightforward and has proven reliability. However we commonly encounter renal injuries that are not explicitly accounted for in the AAST Organ Injury Scale, e.g., renal injuries with segmental devascularization (segmental artery injuries without main renal artery injuries) or multiple cortical lacerations >1 cm in a single renal unit. Such injuries are classified as grade IV at our trauma center; it is these types of grade IV injuries for which we have abandoned routine repeat imaging, and we continue to re-image grade IV injuries with demonstrated urinary extravasation.

## Conclusion

Routine follow-up imaging is unnecessary in the nonoperative management of blunt renal injuries of grades I-III. Grade IV renovascular injuries can be followed clinically without routine follow-up imaging, but urine extravasation necessitates serial imaging to guide management decisions. The volume of grade V renal injuries in this study is not sufficient to support or contest the need for routine follow-up imaging, however we maintain a practice of routine follow-up imaging of nonoperatively managed grade V renal injuries. Ongoing prospective study will test these conclusions.

## Competing interests

The authors declare that they have no competing interests.

## Authors' contributions

JM and ID conceived of the study, participated in design and coordination, and drafted the manuscript. RM and DV participated in study design, data acquisition and analysis. SJ and RG oversaw interpretation of radiographic information. CD and RW helped with manuscript drafting and critical revision. All authors read and approved the final manuscript.

## Pre-publication history

The pre-publication history for this paper can be accessed here:


